# VLDL and LDL Subfractions Enhance the Risk Stratification of Individuals Who Underwent Epstein–Barr Virus‐Based Screening for Nasopharyngeal Carcinoma: A Multicenter Cohort Study

**DOI:** 10.1002/advs.202308765

**Published:** 2024-03-23

**Authors:** Zhenhua Zhou, Tingxi Tang, Nan Li, Qiaocong Zheng, Ting Xiao, Yunming Tian, Jianda Sun, Longshan Zhang, Xiaoqing Wang, Yingqiao Wang, Feng Ye, Zekai Chen, Hanbin Zhang, Xiuting Zheng, Zhen Cai, Laiyu Liu, Jian Guan

**Affiliations:** ^1^ Department of Radiation Oncology Nanfang Hospital Southern Medical University Guangzhou Guangdong China; ^2^ Chronic Airways Diseases Laboratory Department of Respiratory and Critical Care Medicine Nanfang Hospital Southern Medical University Guangzhou Guangdong China; ^3^ Department of Radiation Oncology Yangjiang People's Hospital Yangjiang Guangdong China; ^4^ Department of Radiation Oncology Huizhou People's Hospital Huizhou Guangdong China; ^5^ Department of Radiation Oncology Meizhou People's Hospital Meizhou Guangdong China; ^6^ Department of Laboratory Medicine Nanfang Hospital Southern Medical University Guangzhou Guangdong China; ^7^ Guangdong Province Key Laboratory of Molecular Tumor Pathology Guangzhou Guangdong China

**Keywords:** lipoprotein subfractions, metabolic biomarkers, nasopharyngeal carcinoma, risk stratification

## Abstract

Serological tests for Epstein–Barr virus (EBV) antibodies have been widely conducted for the screening of nasopharyngeal carcinoma (NPC) in endemic areas. Further risk stratification of NPC can be achieved through plasma lipoprotein and metabolic profiles. A total of 297 NPC patients and 149 EBV‐positive participants are enrolled from the NCT03919552 and NCT05682703 cohorts for plasma nuclear magnetic resonance (NMR) metabolomic analysis. Small, dense very low density lipoprotein particles (VLDL‐5) and large, buoyant low density lipoprotein particles (LDL‐1) are found to be closely associated with nasopharyngeal carcinogenesis. Herein, an NMR‐based risk score (NRS), which combines lipoprotein subfractions and metabolic biomarkers relevant to NPC, is developed and well validated within a multicenter cohort. Combining the median cutoff value of the NRS (N_50_) with that of the serological test for EBV antibodies, the risk stratification model achieves a satisfactory performance in which the area under the curve (AUC) is 0.841 (95% confidence interval: 0.811‐0.871), and the positive predictive value (PPV) reaches 70.08% in the combined cohort. These findings not only suggest that VLDL‐5 and LDL‐1 particles can serve as novel risk factors for NPC but also indicate that the NRS has significant potential in personalized risk prediction for NPC.

## Graphical abstract

Comprehensive analysis of lipoprotein and metabolic profiles reveals significant metabolic reprogramming within nasopharyngeal carcinoma (NPC) patients. Small, dense very low density lipoprotein particles (VLDL‐5) and large, buoyant low density lipoprotein particles (LDL‐1) hold promise as novel biomarkers for NPC. The NMR‐based risk score (NRS) demonstrates excellent capability of personalized risk prediction, providing a brand‐new perspective for risk stratification of NPC.

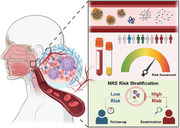

## 1 Introduction

Nasopharyngeal carcinoma (NPC) is a distinct type of highly metastatic and invasive head and neck cancer originating from the nasopharyngeal epithelium, and its incidence is highly variable worldwide. The worldwide incidence of nasopharyngeal carcinoma (NPC) is less than one per 100 000 person‐years, but in endemic regions, including Southeast Asia and southern China, the incidence rate has sharply risen to 20–40 per 100 000 person‐years.^[^ ^1^, ^2^ ^]^ The symptoms of patients with early nasopharyngeal carcinoma are usually insidious and nonspecific, which greatly increases the difficulty of early diagnosis and thus leads to the majority of patients being diagnosed at an advanced stage. Epstein–Barr virus (EBV) infection, genetic susceptibility, environmental factors, and preserved food consumption are generally considered the major carcinogenic factors leading to nasopharyngeal carcinoma.^[^ ^3^ ^]^ A large amount of evidence has supported that EBV infection plays a crucial role in the pathogenesis of NPC, including the detection of EBV DNA and EBV gene expression in precancerous lesions and tumor cells.^[^ ^3^, ^4^ ^]^ Nasopharyngeal carcinoma cells express specific subsets of latent EBV proteins, including Epstein–Barr virus nuclear antigen 1 (EBNA1) and two integrated membrane proteins (latent membrane protein 1 (LMP1) and 2 (LMP2), as well as BamHI‐A fragments of the EBV genome.^[^ ^3^, ^5^ ^]^ Patients with nasopharyngeal carcinoma also show a positive specific serum reaction to many kinds of EBV‐based gene products or antibodies against Epstein–Barr virus. Among these markers, IgA antibodies against EBV capsid antigen (VCA‐IgA) and EBV nuclear antigen 1 (EBNA1‐IgA) are the most frequently used for EBV serological testing in current NPC screening strategies.^[^ ^6^, ^7^, ^8^ ^]^ However, the EBV serological test failed to achieve satisfactory performance largely due to its poor positive predictive value (PPV) of only ≈4%.^[^ ^9^ ^]^ At present, there is still a lack of highly specific and cost‐effective approaches to effectively screen populations and detect high‐risk individuals.

Tumor development and progression are closely related to metabolic reprogramming and lipid metabolism dysregulation, both of which are well‐recognized hallmarks of cancer.^[^ ^10^, ^11^ ^]^ Tumor cells autonomously alter their metabolism and gain adequate nutrition to sustain continuous cell growth and meet increased bioenergetic and biosynthetic demands.^[^ ^12^ ^]^ Several cohort studies have focused on identifying diagnostic biomarkers for NPC at the serum metabolomic level via high‐throughput mass spectrometry. Certain metabolites, including glucose, hydroxyphenylpyruvate, pyroglutamate, and glutamate, which are not specific, have been demonstrated to be potential diagnostic biomarkers for NPC.^[^ ^13^ ^]^ Yi et al. reported that the sensitivity and specificity of seven metabolites (glucose, linoleic acid, stearic acid, arachidonic acid, proline, β‐hydroxy butyrate, and glycerol 1‐hexadecanoate) for NPC diagnosis reached 88.0% and 92.0%, respectively.^[^ ^14^ ^]^ In addition, dysregulated lipid metabolism has increasingly been recognized as a common property of malignant tumors and is highly correlated with the risk of breast, colon, and prostate cancers.^[^ ^15^, ^16^, ^17^ ^]^ Different types of cancer may present different circulating lipoprotein profiles. Liu et al. indicated the potential ability of high‐density lipoprotein (HDL) to promote the proliferation and transformation of NPC cells.^[^ ^18^ ^]^ Although there are no conclusive findings indicating the role of lipoproteins in cancer metabolism, lipoprotein particles or subfractions may be evaluated as potential biomarkers for NPC. Based on these studies and findings, we hypothesized that combining the EBV serological test (VCA/EBNA1‐IgA) with metabolic biomarkers and signature lipoprotein subfractions could improve screening effectiveness and help with personalized risk prediction.

In this study, we innovatively utilized the ^1^H‐Nuclear magnetic resonance (^1^H‐NMR) metabolomic technique, which enables the detection and precise quantification of 38 common metabolites and 112 lipoprotein‐relevant parameters in plasma, to screen signature metabolites and lipoprotein subfractions in a subset of participants (297 patients and 149 controls) from a prospective randomized clinical trial (NCT03919552) and bidirectional clinical trial (NCT05682703). We developed an NMR‐based risk score (NRS) and validated it within an independent, multicenter external validation cohort (84 patients and 53 controls). Our results showed that the median cutoff value of the NRS (N_50_) could achieve a satisfactory performance, as the area under the curve (AUC) was 0.841 (95% confidence interval: 0.811–0.871), and the positive predictive value of screening reached 70.08% in the combined cohort.

Overall, our study not only provides evidence that very low density lipoprotein (VLDL) and low density lipoprotein (LDL) subfractions could serve as novel risk factors for NPC occurrence but also indicates that the NRS along with the EBV serological test could greatly improve screening effectiveness, showing the further potential of carrying out secondary risk stratification to identify high‐risk individuals and adopting personalized follow‐up plans or treatments for populations at different risk levels.

## 2 Results

### 2.1 Study Workflow and Participants’ Demographic Characteristics

**Figure 1:**
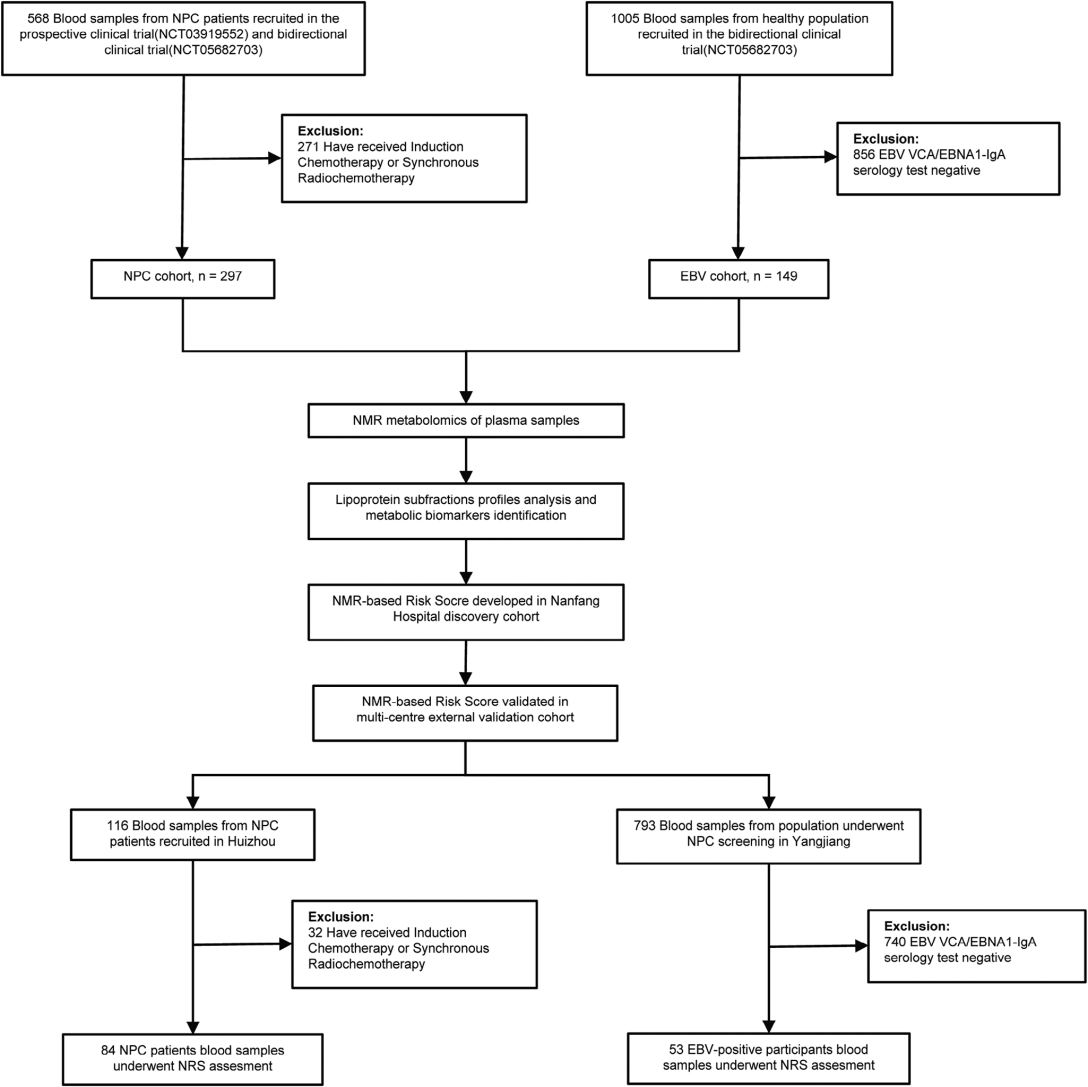
Flowchart of the study.

Between June 2019 and September 2022, 297 NPC patients (NPC cohort) and 149 healthy controls with EBV‐positive serology results (EBV cohort) were enrolled at Nanfang Hospital as the discovery cohort (**Figure** **1**). The demographics and clinical characteristics of all participants are summarized in **Table** **1**. The median ages of the patients in the NPC and EBV cohorts were 52 and 50 years, respectively. The two groups showed similar distributions for body mass index (BMI), with a prevalence of normal weight (18.5<BMI <23.9) in both groups corresponding to 48.48% of the NPC patients and 53.02% of the EBV patients. At diagnosis, only 3 NPC patients were diagnosed at an early stage (stage II, 1.01%), while the remaining patients were diagnosed at an advanced stage (stage III, 28.96%, or stage IV, 70.03%). We also included 84 NPC patients and 53 participants with EBV‐positive serology data from two hospitals in the region with a high incidence of nasopharyngeal carcinoma in Guangdong Province, China (with inclusion and exclusion criteria similar to those of NCT03919552, from June 2019 to September 2022), as an independent multicenter validation cohort to further test the performance of the NRS.

**Table 1:** Clinical and demographic characteristics of the participants.

Characteristic	Discovery Cohort		P value	Validation Cohort		P value
	Case(%)	Control(%)		Case(%)	Control(%)	
	n = 297	n = 149		n = 84	n = 53	
Age						
Median‐yr (IQR)	52(45, 59)	50(36, 58)	< 0.01*	49(44, 56)	48(36, 58)	0.171
<28	8(2.69)	6(4.03)		2(2.38)	4(7.55)	
28‐59	222(74.75)	108(72.48)		65(77.38)	40(75.47)	
>59	67(22.56)	35(23.49)		17(20.24)	9(16.98)	
Sex			0.745			0.977
Male	219(73.74)	112(75.17)		62(73.81)	39(73.58)	
Female	78(26.26)	37(24.83)		22(26.19)	14(26.42)	
BMI						
Median (IQR)	23.5(21, 25.8)	22.7(20.2, 25.4)	0.091	22.2(19.9, 24.6)	21.6(20.1, 22.9)	0.227
<18.5	22(7.41)	16(10.74)		6(7.14)	4(7.55)	
18.5‐23.9	144(48.48)	79(53.02)		55(65.48)	42(79.24)	
>23.9	131(44.11)	54(36.24)		23(27.38)	7(13.21)	
Smoking status			0.463			0.955
Never	104(35.02)	56(37.58)		25(29.76)	17(32.08)	
Current	49(16.50)	18(12.08)		11(13.10)	7(13.21)	
Quit	144(48.48)	75(50.34)		48(57.14)	29(54.72)	
Drinking status			0.625			0.26
Nondrinker	87(29.29)	47(31.54)		14(16.67)	13(24.53)	
Drinker	210(70.71)	102(68.46)		70(83.33)	40(75.47)	
Family history			0.555			0.961
No	279(93.94)	142(95.30)		81(96.43)	52(98.11)	
Yes	18(6.06)	7(4.70)		3(3.57)	1(1.89)	
Stage						
II	3(1.01)	–		1(1.19)	–	
III	86(28.96)	–		19(22.62)	–	
IV	208(70.03)	–		64(76.19)	–	

Statistical methods: Wilcoxon: Age, BMI; Chisq test: Sex, Smoking status, Drinking status, Family history; P value is used to assess the statistical significance of clinical variables between the NPC and EBV cohorts.

**Figure 2:**
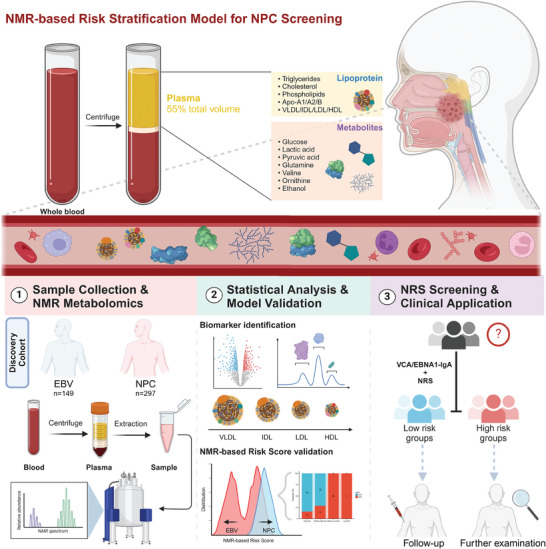
Workflow of the NMR‐based risk score (NRS) construction and its application in NPC screening. The NRS was developed by combining signature metabolites and lipoprotein subfractions relevant to NPC within a discovery cohort from Nanfang Hospital and validated in an independent external validation cohort. The NRS was combined with EBV serology tests for NPC screening.

The study workflow is outlined in **Figure** **2**. Briefly, we conducted nuclear magnetic resonance (NMR) metabolomics analysis on plasma samples from 297 patients and 149 controls via the Bruker IVDr platform. Quantitative analysis of 38 plasma metabolites and 112 lipoprotein subfraction particle‐related indicators was performed. A comprehensive analysis of the characteristic alterations in plasma metabolites and lipoprotein profiles was performed between the NPC and EBV cohorts. Based on orthogonal partial least squares discrimination analysis (OPLS‐DA) and univariate Student's t‐test, we identified several signature metabolites and lipoprotein subfraction particles with high variable importance in projection (VIP > 1) and significant p values (p < 0.05) as potential metabolic biomarkers for NPC. Univariate logistic regression analysis was further employed to identify risk factors closely associated with NPC. Subsequently, we developed a risk stratification model for nasopharyngeal carcinoma based on the NMR‐based risk score (NRS) and validated it within an independent external multicenter validation cohort.

### 2.2 NMR Metabolomics Revealed Alterations in Plasma Metabolite Profiles and Metabolic Reprogramming in the NPC Cohort

We performed nuclear magnetic resonance (NMR) metabolomics analysis on a total of 446 plasma samples from the NPC and EBV cohorts. A total of 38 metabolites and 112 lipoprotein‐related indices were quantitatively measured. We found that the majority of metabolites and lipoprotein components differed significantly between the NPC and EBV cohorts. NPC patients and the EBV population can be effectively distinguished based on the expression profiles of these plasma metabolites and lipoprotein particles (Figure S1A,B, Supporting Information).

**Figure 3:**
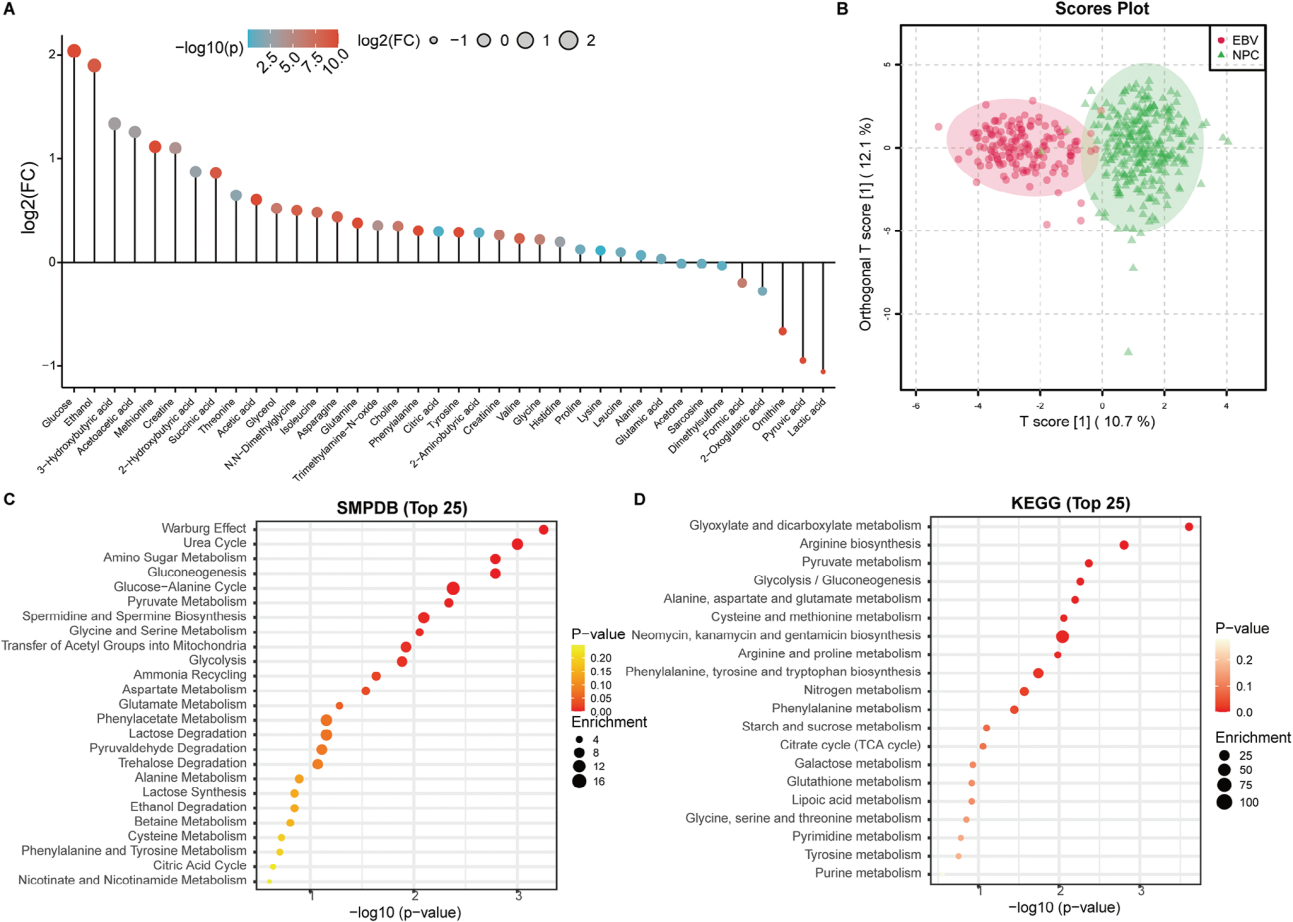
Plasma metabolite profiles and metabolic pathway enrichment analysis in the NPC cohort. A) Differential analysis of 38 plasma metabolites in the NPC and EBV cohorts. FCs were calculated by computing the ratio of the average concentration of each metabolite in the NPC and EBV cohorts. B) Orthogonal PLS‒DA score plot of plasma metabolite profiles of the NPC and EBV cohorts. The abscissa represents the score of the predicted principal component, while the ordinate represents the score of the orthogonal principal component. Overrepresentation analysis of signature metabolites in the SMPDB C) and KEGG D) databases. The size of the circles indicates the number of metabolites enriched in functional pathways.

A comprehensive analysis of 38 common metabolites, including amino acid and glucose metabolism‐related products, was performed (**Figure** **3**A). Our research revealed that the plasma glucose and ethanol levels in NPC patients were significantly greater than those in patients in the EBV group, while the levels of lactic acid and pyruvic acid were significantly lower (Figure S1C, Supporting Information). A total of 8 potential metabolic markers were identified (Figure S1D, Supporting Information). It is well known that tumor cells often possess enhanced glucose uptake and utilization capabilities, implying that they can efficiently absorb glucose from the environment and convert it into energy and other important molecules required for growth.^[^ ^19^, ^20^ ^]^ Enhanced glucose metabolism has been proven to be one of the key characteristics of tumor development and growth processes.^[^ ^12^, ^20^ ^]^ Furthermore, the growth and metabolic activities of tumors themselves can also cause elevated blood glucose levels. For example, tumor cells can produce hormones or cytokines such as insulin resistance factors and corticosteroids, which can interfere with the normal function of insulin, leading to reduced tissue sensitivity to insulin and resulting in elevated blood glucose levels. On the other hand, tumor growth may lead to a persistent stress state in the body, releasing stress hormones such as cortisol and adrenaline. These hormones can promote glycogenolysis in the liver, increasing blood glucose levels.^[^ ^21^ ^]^

Utilizing the OPLS‐DA algorithm, the results showed that the NPC and EBV cohorts could be effectively distinguished via the plasma metabolite profiles (Figure 3B). Metabolite set enrichment analysis (MSEA) was performed on the NPC and EBV cohorts, and the results confirmed the occurrence of significant metabolic reprogramming among NPC patients. Notably, in addition to the enrichment of most glycolytic and amino acid metabolism pathways, sphingolipid metabolism was highly enriched (Figure S2A–D, Supporting Information). Sphingolipids play a crucial role in lipid transport by acting as carrier molecules involved in the transport and metabolic regulation of cholesterol and lipids, thereby maintaining lipid balance. These findings suggest the potential presence of significant lipid metabolism disorders in the NPC cohort.

We screened out metabolites that significantly contributed to distinguishing between nasopharyngeal carcinoma patients and the EBV‐infected population (VIP > 1, p < 0.05), including glucose, lactic acid, pyruvic acid, glutamine, methionine, acetic acid, phenylalanine, and ornithine (Figure S1D, Supporting Information). Enrichment analysis of these metabolites indicated that metabolic disorders related to glucose metabolism, such as the Warburg effect, pyruvate metabolism, and glycolysis, were significantly different between nasopharyngeal carcinoma patients and the EBV cohort, which was consistent with several previous studiesx (Figure 3C,D).^[^ ^12^, ^20^, ^22^ ^]^

### 2.3 VLDL and LDL Subfractions Promote the Occurrence of NPC

**Figure 4:**
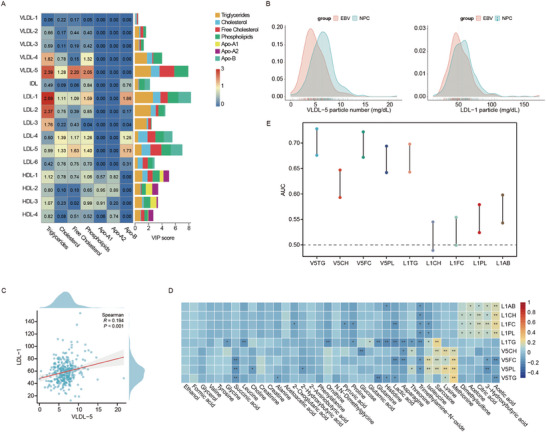
The heterogeneity of lipoprotein subfraction particles and correlations with plasma metabolites. A) Variable importance in projection (VIP) of lipoprotein subfraction particles in the discovery cohort. The VIP score refers to the sum of the VIPs of each component of the lipoprotein particles. B) Density plot showing the distribution of VLDL‐5 and LDL‐1 particle numbers between the NPC and EBV cohorts. The dotted line represents the median value of the number of particles. C) Spearman correlation analysis of LDL‐1 and VLDL‐5 particles in the NPC cohort. The R‐value represents the correlation coefficient between variables in the sample. D) Correlation analysis of LDL‐1 and VLDL‐5 levels with 38 plasma metabolites. The color bar represents the correlation coefficient. ^*^, p < 0.05; ^**^, p < 0.01. E) Dumbbell diagram showing the area under the receiver operating characteristic curve (AUROC) of the VLDL‐5 and LDL‐1 particle components in the discovery dataset. The error bars represent the 95% confidence intervals. TG, triglycerides; CH, cholesterol; FC, free cholesterol; PL, phospholipids; AB, apolipoprotein‐B.

The ^1^H‐NMR plasma lipoprotein profile included a total of 112 lipoprotein parameters (Table S1, Supporting Information). There were significant differences in the blood lipoprotein profiles between the NPC and EBV cohorts, primarily involving the VLDL and LDL components. Orthogonal partial least squares discrimination analysis (OPLS‐DA) indicated that the distribution of NPC patients was significantly different from that of patients in the EBV group (Figure S3A–C, Supporting Information). We compared the levels of traditional lipoprotein particles between the NPC and EBV cohorts using the Mann–Whitney U test, which indicated that there were significant differences in the number of VLDL and LDL particles (Figure S3D, Supporting Information). The lipoprotein subfraction particles that significantly contributed to group separation (VIP>1) were mainly VLDLs and LDLs (**Figure** **4**A).

Further subgroup analysis of the lipoprotein profiles demonstrated that all structural components of the VLDL‐5 (triglycerides,TG; cholesterol, CH; free cholesterol, FC; phospholipids, PL) and LDL‐1 (triglycerides, TG; cholesterol, CH; free cholesterol, FC; phospholipids, PL; apolipoprotein‐B, Apo‐B) particles had significant VIP values (VIP > 1), suggesting a close association between these two lipoprotein subfractions and the occurrence of NPC (Figure 4A). We conducted further analysis to compare the differences in VLDL‐5 and LDL‐1 particles between the NPC and EBV groups. The distribution of VLDL‐5 and LDL‐1 particles in the plasma of NPC patients resembled that in the plasma of patients in the EBV group but was significantly greater (Figure 4B). Spearman correlation analysis of LDL‐1 and VLDL‐5 levels in the NPC cohort indicated almost no correlation between the two variables (Figure 4C). This suggested that the synthesis, assembly, and metabolism of LDL‐1 and VLDL‐5 particles are likely to be independent of each other and may serve as independent risk factors closely associated with the development of NPC.

To further explore the roles of LDL‐1 and VLDL‐5 particles in the process of nasopharyngeal carcinoma development, we performed a correlation analysis between the structural components (TG, CH, FC, PL, Apo‐B) of these two particles and plasma metabolomic profiles. Notably, the structural components of LDL‐1 particles showed positive correlations mainly with molecules involved in carbohydrate metabolism, such as acetic acid, 3‐hydroxybutyric acid, citric acid, and acetone. On the other hand, the structural components of the VLDL‐5 particles exhibited positive correlations primarily with molecules involved in amino acid metabolism, such as methionine, lysine, sarcosine, and isoleucine (Figure 4D). We hypothesized that LDL‐1 particles may participate in the disruption of carbohydrate metabolism to provide energy support for tumor growth, while VLDL‐5 particles are more likely involved in the regulation of amino acid metabolism to provide nutritional support for tumor growth (Figure S4A–D, Supporting Information). Although the association between LDL and VLDL subfraction particles and carbohydrate and amino acid metabolism has rarely been reported, our study supported the notion that LDL‐1 and VLDL‐5 particles are likely to directly or indirectly participate in carbohydrate or amino acid metabolic pathways, thus promoting the development of NPC. The specific underlying mechanisms still require further exploration.

We also conducted ROC analysis on the structural components of LDL‐1 and VLDL‐5 in our discovery dataset. The results showed that several components of VLDL‐5 and LDL‐1 achieved satisfactory AUC values with an average greater than 0.6, which demonstrated the great potential of VLDL‐5 and LDL‐1 particles, along with their associated structural components, to serve as biomarkers for nasopharyngeal carcinoma (Figure 4E).

### 2.4 Construction of the NMR‐Based Risk Stratification Model and Its Clinical Application Combined with the EBV Serology Test

**Figure 5:**
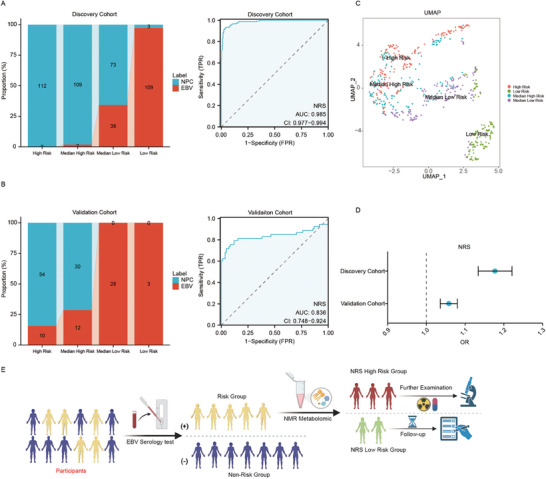
A risk stratification model for nasopharyngeal carcinoma based on NMR‐based risk score and its clinical application combined with EBV serology test. A) NRS risk stratification model of the discovery cohort (left) and ROC analysis of the NRS in the discovery cohort (right); B) NRS risk stratification model of the validation cohort (left) and ROC analysis of the NRS in the validation cohort (right); C) UMAP analysis of signature metabolites and lipoprotein subfraction particle profiles of the combined cohort. D) Odds ratios (ORs) of the NRS score in the discovery and validation cohorts. E) The NMR‐based risk score (NRS) combined with the EBV serology test can aid in personalized risk prediction and accurate identification of high‐risk individuals for the early diagnosis of NPC.

Based on the discovery dataset, a total of 8 plasma signature metabolites and 2 lipoprotein subfraction particles (LDL‐1 and VLDL‐5) were selected as candidate factors for the risk stratification model. The high and low groups were distinguished according to the median of the quantitative data of each factor, and univariate logistic regression analysis was performed on the other candidate factors (**Table** **2**). Ultimately, 5 metabolites (glucose, lactic acid, pyruvic acid, methionine, ornithine), 4 components of VLDL‐5 particles (triglycerides, cholesterol, free cholesterol, and phospholipids) and 3 components of LDL‐1 particles (triglycerides, phospholipids, and Apo‐B), which are closely related to NPC carcinogenesis, were identified as modeling factors. An NMR‐based risk score (NRS) was constructed for all participants according to quantitative data and OR values of modeling factors (Methods). Taking the top 25th percentile (N_25_), median value (N_50_) and top 75th percentile (N_75_) of the NRS as thresholds, all participants in the discovery cohort were divided into a high‐risk group (NRS > N_25_), a medium‐high‐risk group (N_50_ < NRS < N_25_), a medium‐low‐risk group (N_75_ < NRS < N_50_) and a low‐risk group (NRS < N_75_). The number of NPC patients and EBV‐infected individuals in each risk stratification group was counted. In the discovery cohort, we found that all individuals in the high‐risk group were NPC patients, while only 2 EBV‐infected individuals (1.3%) were classified into the medium‐high‐risk group. In the low‐risk group, there were only 3 NPC patients (1.0%), while 73 NPC patients (24.6%) were classified into the medium‐low‐risk group (**Figure** **5**A). Receiver operating characteristic (ROC) analysis of the NRS in the discovery cohort yielded an AUC of 0.985 (95% CI: 0.977–0.994) (Figure 5A). To further validate the efficacy of the NRS risk stratification model, we tested it within an independent multicenter external validation cohort (n = 137). In the validation cohort, all individuals in the low‐risk and medium‐low‐risk groups were EBV‐infected, while only 10 individuals (18.9%) and 12 individuals (22.6%) in the EBV‐infected groups were classified into the high‐risk and medium‐high‐risk groups, respectively. Similarly, receiver operating characteristic (ROC) analysis revealed that the area under the curve (AUC) of the NRS in the validation cohort reached 0.836 (95% CI: 0.748–0.924) (Figure 5B).

**Table 2:** Univariate logistic regression analysis of signature metabolites and lipoprotein subfractions in the discovery cohort.

Characteristics	Median value	Group	OR [95% CI]	P value
Signature metabolites	(mmol L^−1^)			
Glucose	3.7835	Low	Reference	
		High	139.048(43.059–449.015)	<0.01*
Lactic acid	5.3065	Low	Reference	
		High	0.002(0–0.017)	<0.01*
Pyruvic acid	0.228	Low	Reference	
		High	13.37(7.928–22.547)	<0.01*
Glutamine	0.5545	Low	Reference	
		High	1.3(0.876–1.929)	0.192
Methionine	0.01	Low	Reference	
		High	4.339(2.814–6.691)	<0.01*
Acetic acid	0.009	Low	Reference	
		High	1.475(0.989–2.199)	0.057
Phenylalanine	0.048	Low	Reference	
		High	0.879(0.593–1.304)	0.523
Ornithine	0.041	Low	Reference	
		High	0.074(0.044–0.124)	<0.01*
Lipoprotein subfractions	(mg dL^−1^)			
VLDL‐5 Triglycerides	2.245	Low	Reference	
		High	3.975(2.596–6.088)	<0.01*
VLDL‐5 Cholesterol	1.44	Low	Reference	
		High	2.732(1.81–4.124)	<0.01*
VLDL‐5 Free Cholesterol	0.45	Low	Reference	
		High	3.919(2.56–6.002)	<0.01*
VLDL‐5 Phospholipids	1.72	Low	Reference	
		High	3.257(2.145–4.945)	<0.01*
LDL‐1 Triglycerides	4.82	Low	Reference	
		High	3.52(2.309–5.365)	<0.01*
LDL‐1 Cholesterol	21.165	Low	Reference	
		High	1.249(0.842–1.852)	0.27
LDL‐1 Free Cholesterol	6.21	Low	Reference	
		High	1.449(0.975–2.153)	0.066
LDL‐1 Phospholipids	11.91	Low	Reference	
		High	1.731(1.162–2.579)	<0.01*
LDL‐1 Apo‐B	11.125	Low	Reference	
		High	1.881(1.261–2.806)	<0.01*

We performed UMAP dimensionality reduction analysis on the signature metabolite and lipoprotein subfraction profiles of participants in different risk groups from the discovery and validation cohorts. The results showed distinct separation among the four risk groups (Figure 5C). Additionally, univariate logistic regression analysis of the NRS score in the discovery and validation cohorts revealed that the NRS score could serve as an independent risk factor for NPC, with an odds ratio (OR) of 1.177 (95% CI: 1.134–1.221) in the discovery cohort and an odds ratio (OR) of 1.058 (95% CI: 1.036–1.08) in the validation cohort (Figure 5D).

To evaluate the potential of the NRS for NPC screening, we combined the NRS with serum EBV VCA/EBNA1‐IgA tests for discriminating NPC patients and calculated the specificity, sensitivity, PPV, NPV, and Youden index based on these tests (Methods). We combined the NRS at various cutoff values with the EBV serology test results and classified only individuals who were identified as high risk by both the NRS > cutoff value and the EBV serology test as the high‐risk group. In the combined cohort, when the top 50th percentile of the NRS (N_50_) was used as the cutoff value, the AUC of the combined NRS (0.841, 95% CI: 0.811–0.871) was much greater than that of the top 25th percentile (N_25_) (0.693, 95% CI: 0.664–0.722) or 75th percentile (N_75_) (0.773, 95% CI: 0.739–0.808), indicating that the NRS risk stratification model had the best performance, with both specificity and sensitivity reaching over 0.80 (PPV: 0.7008; NPV: 0.9271; Youden index: 0.6817) (**Table** **3**).

**Table 3:** Prediction performance of the NRS with different cutoff values in the combined cohort.

Model	Cut‐off	AUC [95%CI]	Specificity	Sensitivity	PPV	NPV	Youden index
NRS(Top 25 percentile)	NRS>38.97	0.693 (0.664–0.722)	0.4357	0.9505	0.4717	0.9432	0.3862
NRS(Top 50 percentile)	NRS>14.95	0.841 (0.811–0.871)	0.8005	0.8812	0.7008	0.9271	0.6817
NRS(Top 75 percentile)	NRS>−20.21	0.773 (0.739–0.808)	0.9921	0.5545	0.9739	0.8077	0.5466

In our study, the NRS risk stratification model based on plasma NMR metabolomics could effectively discriminate NPC patients from EBV‐infected individuals, achieving more accurate identification of high‐risk individuals with NPC. Combining the median cutoff value of the NRS (N_50_) with that of the EBV serology test, the positive predictive value of screening reached 70% in the combined cohort (Figure 5E).

These results indicate that implementing the NRS as an addition to standard EBV VCA/EBNA1‐IgA serology tests substantially enhances the PPV for determining NPC in an endemic southern Chinese population.

## 3 Discussion

The prevalence of NPC shows notable regional variations, with the highest occurrence observed in southern China and Southeast Asia. In the southern region of China, statistics indicate that the incidence rate of NPC among males is ≈30 cases per 100 000 individuals, which is ≈2.3 times that of females. Notably, China alone contributes 47% of the global annual NPC cases.^[^ ^1^, ^2^ ^]^ Improving the prevention of NPC has increasingly become one of the major public health issues in China, especially in endemic regions. Herein, we innovatively report an effective, easy, and affordable risk stratification model based on plasma signature metabolites and lipoprotein subfractions that could more accurately distinguish NPC patients from Epstein–Barr virus‐positive individuals (AUC: 0.841, PPV: 0.7008, specificity: 0.8005, sensitivity: 0.8812), suggesting a promising prospect for secondary risk stratification of NPC patients.

Our study presented original findings on the distinctive alterations in plasma lipoprotein profiles of patients with nasopharyngeal carcinoma (NPC) and identified specific lipoprotein subfraction particles that are highly important for the risk stratification of NPC. Notably, we found that small dense VLDL particles (VLDL‐5) and large buoyant LDL particles (LDL‐1) have promising potential as effective biomarkers for monitoring the occurrence of nasopharyngeal carcinoma, which has rarely been reported in the field of NPC research. VLDL is metabolized to LDL after secretion, and LDL that enters arterial walls can be oxidized by vascular cells (endothelial cells, smooth muscle cells, and macrophages) using oxidases (including lipoxygenase and myeloperoxidase). The greater the amount of TG and cholesteryl ester (CE) carried by lipoproteins from VLDL metabolism, the greater the risk of oxidation.^[^ ^23^ ^]^ Previous studies have shown that LDL‐1 and VLDL subfractions can promote breast cancer metastasis and progression through Akt‐induced epithelial‐mesenchymal transition (EMT) and angiogenesis.^[^ ^24^ ^]^ The type II VLDL receptor in lipoprotein receptors suggests a potential link to tumor invasion and metastasis.^[^ ^25^ ^]^

We established a foundation for understanding the relationship between the occurrence of nasopharyngeal carcinoma and the presence of lipoprotein subfraction particles and postulated that lipid‐lowering therapies targeting VLDL or LDL subfraction particles may have broader clinical implications for reducing the incidence of nasopharyngeal carcinoma or inhibiting tumor progression; more clinical trials are urgently needed to validate these findings in the future. Interestingly, several studies investigating the role of lipoprotein particles in tumor development have yielded inconsistent findings. Zhang et al. suggested that low serum total cholesterol (TC) levels independently increase the risk of gastric cancer, with gastric cancer patients exhibiting significant decreases in serum TC, HDL cholesterol (HDL‐C), and LDL cholesterol (LDL‐C) compared to healthy individuals. However, the levels of lipoprotein(a) increase with the malignant stage of gastric cancer.^[^ ^26^ ^]^ Research on ovarian cancer has shown that blood lipid TC and HDL‐C levels gradually decrease with tumor progression. The measurement of these two indicators plays an important clinical role in the diagnosis and differentiation of benign and malignant ovarian tumors.^[^ ^27^ ^]^ Hyperlipidemia was closely associated with the risk of breast cancer, as increased LDL‐C indicated an elevated likelihood of axillary lymph node metastasis in breast cancer patients, while TC and TG were found to be independent high‐risk factors for distant metastasis in breast cancer patients.^[^ ^28^, ^29^ ^]^ Nonetheless, these studies have consistently demonstrated the close relationship between lipid metabolism and various malignant tumors, highlighting the significance of monitoring changes in blood lipid metabolism and controlling lipid levels for assessing disease progression and prevention in cancer patients.

The findings also indicated that elevated plasma glucose levels and reduced plasma lactic acid levels may be potential indicators for NPC, although they are not specific. The hyperglycemic microenvironment not only provides the necessary nutrients and energy for tumor cell growth but also potentially enhances tumor cell proliferation and metastatic ability.^[^ ^20^ ^]^ Additionally, lactic acid, the end product of glycolysis, is implicated in facilitating tumor growth and metastasis and is associated with poor prognosis. Lactic acid can also be utilized in transamination reactions to generate alanine, promoting tumor protein synthesis and accelerating tumor growth. Notably, recent research has revealed an association between high serum lactate levels and adverse prognostic outcomes, including overall survival, disease‐free survival, and metastasis‐free survival, in patients with cervical cancer, head and neck tumors, high‐grade glioma, and non‐small cell lung cancer.^[^ ^30^, ^31^, ^32^, ^33^, ^34^, ^35^, ^36^ ^]^ Conversely, elevated lactic acid levels (>5.3065 mmol L^−1^) in plasma were considered a protective factor (OR: 0.002, p < 0.01) against NPC in our study. The protective value of elevated lactic acid should be further explored in basic and clinical research.

Our NRS model is a secondary risk stratification tool based on EBV antibody serology, which not only effectively compensates for the limitations of traditional EBV‐based screening methods for nasopharyngeal carcinoma but also demonstrates the significant role of metabolomics in monitoring the occurrence and progression of tumors. Efforts to develop early detection screening for NPC have primarily concentrated on EBV‐based tests, which involve examining serum anti‐EBV antibodies and plasma EBV DNA. A logistic regression model has been developed to predict NPC using a combined score of viral capsid antigen and Epstein–Barr nuclear antigen 1 (VCA/EBNA1) immunoglobulin A (IgA) antibodies, which exhibited a specificity of 97% and a positive predictive value (PPV) of 4.4% in a large cluster randomized trial (NCT‐00941538) within a 1‐year follow‐up period.^[^ ^6^ ^]^ Plasma EBV DNA testing for NPC screening was prospectively evaluated in an observational trial involving 20174 middle‐aged men (aged 40–62 years). The 2‐time point testing protocol exhibited a positive predictive value (PPV) of 11%.^[^ ^37^ ^]^ Although several approaches showed satisfactory sensitivity or specificity, poor positive predictive value (PPV) and accuracy, along with the lack of unified criteria in testing machines, reagents, methods, and other techniques among units and centers, a relatively low proportion of early‐stage nasopharyngeal carcinoma patients were diagnosed.

Combining the NMR‐based risk score (NRS) with the EBV VCA/EBNA1‐IgA serology test, this novel screening strategy for nasopharyngeal carcinoma not only reveals promising application for the identification of high‐risk individuals with EBV‐infected populations but also leads to a substantial reduction in the financial burden and unnecessary medical examinations for participants. Our approach demonstrates excellent cost‐effectiveness and enhances the effectiveness of secondary risk stratification. In our combined cohort, individuals with positive EBV VCA/EBNA1‐IgA serology results were identified as potential risk groups for NPC. Within this risk group, risk stratification based on the NRS score was performed using the median cutoff NRS score (N_50_). The findings revealed that a total of 24 individuals were classified into the NRS high‐risk group, while 178 individuals were categorized into the NRS low‐risk group. For individuals in the NRS high‐risk group, we recommend prompt completion of nasopharyngeal endoscopic examination and magnetic resonance imaging (MRI), along with increased follow‐up frequency, to dynamically monitor their clinical symptoms and examination results (e.g., reassessing NRS risk stratification results within 3–6 months or conducting additional necessary investigations). Regarding individuals in the NRS low‐risk group, additional investigations for further nasopharyngeal carcinoma screening are unnecessary. Instead, we propose regular blood monitoring of NRS risk stratification results every 6–12 months. If certain individuals are reclassified to the NRS high‐risk group during follow‐up, further examinations, such as nasopharyngeal endoscopic examination or magnetic resonance imaging (MRI), are recommended for screening for nasopharyngeal carcinoma (NPC).

For health economics assessment, the cost of plasma nuclear magnetic resonance metabolomics testing was ≈$30 per person, while the cost of nasopharyngeal endoscopic examination or magnetic resonance imaging (MRI) ranged from 70 to $200 in our study. This two‐step screening strategy identified 12% of the EBV‐infected population as the NRS high‐risk group, potentially benefiting from additional preventive measures or monitoring to facilitate early diagnosis at an appropriate age. Moreover, the remaining ≈88% of the general EBV‐infected population can avoid unnecessary medical examinations, thus reducing the economic burden.^[^ ^38^ ^]^ Considering its relatively low cost and high positive predictive value (PPV), the combination of the NRS and EBV serology test is a promising and cost‐effective tool for risk discrimination, clinical decision‐making, and efficient resource allocation.

The aim of this multicenter, cross‐sectional study was to utilize metabolomics techniques to screen for metabolic biomarkers and lipoprotein subfractions that could monitor the occurrence of nasopharyngeal carcinoma (NPC) and ultimately construct a personalized risk assessment model based on the NRS. Our NRS achieved satisfactory results in a multicenter, external validation cohort and demonstrated excellent performance according to the NRS threshold (N_50_) for risk stratification in the combined cohort. We will further validate these potential NPC biomarkers in additional clinical cohorts. Moreover, prior to implementing the NRS in population screening and counseling programs, we intend to conduct further assessments of its performance through larger‐scale, cross‐regional, multicenter, prospective clinical studies. Nonetheless, this study has several limitations. For instance, LDL‐1 and VLDL‐5 were identified as potential carcinogenic particles associated with NPC and closely linked to glucose and amino acid metabolism. However, the specific roles these particles play in these metabolic pathways and the molecular mechanisms through which they contribute to cancer promotion remain unclear. Further in‐depth basic research is needed to explore and validate these aspects. Additionally, the present study revealed significant changes in signature metabolites, particularly glucose‐related products, and LDL‐1/VLDL‐5 lipoprotein subfractions in the NPC population. However, whether interventions such as glucose‐lowering or lipid‐lowering measures can effectively prevent the occurrence of nasopharyngeal carcinoma still requires further exploration in prospective clinical studies and long‐term follow‐up observations.

In conclusion, we developed an NRS risk prediction model utilizing plasma metabolites and lipoprotein particles for precise risk stratification of NPC patients. This NRS model not only enables personalized risk assessment for nasopharyngeal carcinoma in southern China but also holds potential for broader application in larger‐scale, cross‐regional, and even cross‐ethnic populations for risk monitoring. The integration of the NRS with the current standard EBV serology‐based screening method significantly enhances the accuracy of nasopharyngeal carcinoma screening and has favorable economic benefits. This study is of paramount importance in establishing effective screening and early diagnosis strategies for individuals at high risk of nasopharyngeal carcinoma.

## 4 Experimental Section

### Participants and Study Design

All participants in the discovery cohort were derived from two independent clinical trial cohorts. NCT03919552 was a prospective, randomized, double‐blind, multicenter phase III clinical trial conducted at Nanfang Hospital in June 2019. The aim of this study was to investigate the differences in the efficacy and long‐term toxicity of cisplatin and carboplatin in combination with concurrent chemotherapy for patients with locally advanced nasopharyngeal carcinoma. The inclusion criteria mainly included pathologically confirmed nonkeratinizing nasopharyngeal carcinoma (WHO type II or III), AJCC stage T3‐4NxM0/TxN2‐3M0, and no prior radiotherapy or treatment. Male or nonpregnant female patients aged 18–65 years with Karnofsky Performance Scale (KPS) scores ≥70 and normal bone marrow, liver, and kidney function were eligible. Patients with a history of malignancy, other severe diseases, including unstable cardiac disease, renal disease, abnormal liver function, poorly controlled diabetes mellitus (fasting blood glucose >1.5 times the normal value), mental illness, or severe allergic history, were excluded to better evaluate the efficacy of chemotherapy. To date, more than 200 participants have been recruited into this clinical trial. Complete blood samples, plasma samples, serum samples, and epidemiological characteristics of each patient were collected and stored at different stages of treatment, and all clinical procedures were performed with the patient's fully informed consent. The NCT05682703 trial was a bidirectional study to explore the dynamic changes in plasma and urine metabolites during the occurrence and development of nasopharyngeal carcinoma in southern China. This was a multicenter, bidirectional, two‐arm, 3‐year observational cohort study. The study population consisted mainly of the EBV group, which included individuals whose EBV VCA/EBNA1‐IgA serology test was positive; the NPC group, which included patients who were pathologically diagnosed with NPC (2018 WHO criteria); and the healthy group, which included healthy people who had been hospitalized in the physical examination center of Nanfang Hospital without a history of nasopharyngeal‐related diseases or other known diseases that may affect blood lipid/protein metabolism. All clinical studies were conducted under the supervision of the ethics committee of Southern Medical University Nanfang Hospital.

Ultimately 297 nasopharyngeal carcinoma patients (NPC cohort) and 149 healthy controls with EBV infection (EBV cohort) as the discovery cohort were included. The patients from three hospitals in the region with a high incidence of nasopharyngeal carcinoma in Guangdong Province, China (with inclusion and exclusion criteria similar to those used for NCT03919552, from June 2019 to September 2022), and healthy individuals with positive EBV serology test results to further test the performance of the NRS in this multicenter external validation cohort were also included. The study flowchart is shown in Figure 1.

### Sample Collection and Processing

Peripheral blood from Nanfang Hospital was collected using a serum collection tube (BD Biosciences, San Jose, USA). All blood specimens were collected following a standardized collection process. Blood samples were obtained using anticoagulant tubes containing sodium heparin. Plasma was separated within 24 h using a centrifuge at 2000 rpm for 10 min at 4 °C. A total of 1000 µL of each tube was aliquoted and stored at ultralow temperatures (−80 °C) using cryopreservation techniques. The plasma in the supernatant was transferred to clean cryovials and stored at −80 °C until ^1^H‐NMR analysis. To ensure the stability of metabolites and preserve their original state, dry ice courier services were employed during shipment and transportation to maintain low temperatures.

### ^1^H‐NMR Metabolite and Lipoprotein Profiles

A total of 38 endogenous metabolites in plasma were measured using 1D proton (^1^H) nuclear magnetic resonance (NMR) spectroscopy. All plasma samples were analyzed following the standard operating procedures developed by the laboratory. Initially, the plasma samples were thawed after being removed from the refrigerator. Then, 400 µL of each plasma sample was mixed with an equal volume of NMR lipid buffer (Bruker Biospin, Ettlingen, Germany) at a 1:1 ratio. The mixture was homogenized by vortexing for 30 s and transferred to a 5 mm NMR tube for analysis. ^1^H‐NMR spectra were obtained at 310 K using a Bruker 600 MHz NMR spectrometer operated at a proton Larmor frequency of 600.13 MHz. To perform lipoprotein subfraction analysis and lipid quantification, the laboratory utilized the Bruker IVDr Lipoprotein Subclass Analysis platform (B.I.‐LISA, Bruker BioSpin, Singapore). This platform employs a validated prediction algorithm based on the partial least squares (PLS) regression model. The lipoprotein panel included various fractions, such as total HDL, LDL, IDL, and VLDL, along with 15 subfractions sorted numerically by increasing density and decreasing size: 4 HDL subfractions (HDL‐1–HDL‐4), 6 LDL subfractions (LDL‐1–LDL‐6), and 5 VLDL subfractions (VLDL‐1–VLDL‐5). The specific density ranges for each lipoprotein subfraction are listed in Table S2 (Supporting Information). Each subfraction was quantified for parameters such as cholesterol, free cholesterol, phospholipids, triglycerides, Apo A1, A2, and B100 fractions, as well as 9 particle numbers. Additionally, ratios such as Chol‐LDL/CholHDL and Apo‐B100/Apo‐A1 were calculated, resulting in a total of 112 parameters (Figure S4, Supporting Information).

### NMR‐Based Risk Score Construction and Validation

In the discovery cohort, a total of 8 signature metabolites (glucose, lactic acid, pyruvic acid, glutamine, methionine, acetic acid, phenylalanine, ornithine) and 2 subfractions of lipoprotein particles (VLDL‐5, LDL‐1) were identified by comparing the nuclear magnetic resonance metabolomics profiles of plasma between the NPC and EBV cohorts. Subsequently, univariate logistic regression analysis on the signature metabolites and lipoprotein subfraction components in the discovery cohort was performed. It was determined that 5 metabolites (glucose, lactic acid, pyruvic acid, methionine, ornithine), 4 components of the VLDL‐5 particle (triglycerides, cholesterol, free cholesterol, and phospholipids), and 3 components of the LDL‐1 particle (triglycerides, phospholipids, and Apo‐B) were highly correlated risk factors for nasopharyngeal carcinoma (NPC). These factors were then incorporated into the risk stratification model for NPC.

The NMR‐based risk score (NRS) was derived for each individual using the following formula:

(1) $$\sum_{i=1}^{n}x_{i}\ln\left(\beta_{i}\right)$$

where x is the value of an individual risk factor and β is the odds ratio (OR) for the specific risk factor. The effect size of each risk factor (β) was derived from a logistic regression analysis of the discovery dataset. A receiver operating characteristic (ROC) curve analysis was performed to assess the performance of the NMR‐based risk score (NRS) in discriminating NPC patients from controls. Finally, logistic regression models were used to estimate the odds ratio (OR) for NPC in relation to the NRS score in the discovery and external validation cohorts.

### Statistical Analysis

For the discovery cohort, NMR metabolite and lipoprotein subfraction particle profiles were analyzed by MetaboAnalyst V5.0 (<https://www.metaboanalyst.ca/>). MetaboAnalyst is a comprehensive platform dedicated to metabolomics data analysis via a user‐friendly, web‐based interface. The current MetaboAnalyst V5.0 supports raw MS spectra processing, comprehensive data normalization, statistical analysis, functional analysis, meta‐analysis, and integrative analysis with other omics data. The metabolite profiles and lipoprotein profiles were uploaded to the Statistical Analysis [one factor] module for data processing and normalization, respectively. Data processing contains the following processes in sequence: Reading Concentration Data, Data Integrity Check, Missing Value Imputations, and Data Filtering. All the data were numeric, 1 feature with a constant or single value across samples was found and deleted, and a total of 0 (0%) missing values were detected. The data were stored as a table with one sample per row and one variable (bin/peak/metabolite) per column. The normalization procedures implemented below were grouped into four categories. Sample‐specific normalization allows users to manually adjust concentrations based on biological inputs (i.e., volume, mass); row‐wise normalization allows general‐purpose adjustment for differences among samples; and data transformation and scaling are two different approaches to make features more comparable. The raw data were subjected to standard normalization procedures as follows: row‐wise procedures: normalization by the sum; data transformation: log transformation (base 10); and data scaling: auto‐scaling (mean‐centered and divided by the standard deviation of each variable).

Orthogonal PLS‐DA analysis was conducted to determine differences in metabolite and lipoprotein subtraction parameters between the NPC and EBV cohorts. The quality parameters R^2^ and Q^2^ were used to evaluate the goodness of fit and the predictive ability of the model. Significantly different lipoprotein subfraction particles and signature metabolites were selected by variable importance in projection (VIP>1) and univariate Student's t‐tests (p<0.05). Over representation analysis of signature metabolites was performed by the Enrichment Analysis module in MetaboAnalyst V5.0, in which SMPDB (99 metabolite sets based on normal human metabolic pathways) and KEGG (84 metabolite sets based on KEGG human metabolic pathways) were selected as pathway‐based metabolite set libraries.

Univariate logistic regression analysis of signature metabolites and lipoprotein subfraction particles in the discovery cohort was performed using IBM SPSS Statistics 26.0. The pROC package version 1.18.0 was used to estimate the performance of the NRS risk stratification model in classifying NPC groups versus EBV groups and to calculate the sensitivity, specificity, PPV, NPV, and Youden index. PPV = TP/(TP+FP); NPV = TN/(TN+FN); Youden index = sensitivity + specificity – 1; TP: true positive; FP: false‐positive; TN: true negative; FN: false‐negative.

### Ethics Approval Statement

The research conducted for this study was based on clinical trials(NCT03919552 and NCT05682703), which have been conducted in accordance with ethical principles and guidelines. The study protocol and research activities have received approval from the ethics committee of Southern Medical University Nanfang Hospital for the commencement of the study. All human subjects involved in the study provided informed consent before their inclusion in the research. The study protocol adheres to the principles outlined in the Declaration of Helsinki and applicable national and international regulations and guidelines.

## Conflict of Interest

The authors declare no conflict of interest.

## Author Contributions

Z.Z., T.T., and N.L. contributed equally to this work. J.G. and Z.Z. performed conceptualization. Z.Z., T.T., N.L., and J.G. performed methodology. Z.Z., T.T., N.L., Q.Z., T.X., Y.T., J.S., L.Z., X.W., Y.W., F.Y., Z.C., H.Z., X.Z., Z.C., L.L., and J.G. performed Investigation. J.G. and Z.Z. performed project administration. J.G. performed supervision. J.G and Z.Z. wrote the original draft. J.G., Z.Z., T.T., and N.L. reviewed and edited the manuscript.

## Supporting information

Supporting Information

Supporting Information

## Data Availability

The data that support the findings of this study are available on request from the corresponding author. The data are not publicly available due to privacy or ethical restrictions.
