# Supplementary material for: VLDL and LDL Subfractions Enhance the Risk Stratification of Individuals Who Underwent Epstein–Barr Virus‐Based Screening for Nasopharyngeal Carcinoma: A Multicenter Cohort Study
Source: Adv Sci (Weinh). 2024 Mar 23;11(22):2308765. doi: 10.1002/advs.202308765 (PMC11165512; doi:10.1002/advs.202308765)
Supplement: Supplementary file 1 — Supporting Information [file ADVS-11-2308765-s001.pdf]

ADVANCED SCIENCE
Open Access

Supporting Information

for *Adv. Sci.*, DOI 10.1002/advs.202308765

VLDL and LDL Subfractions Enhance the Risk Stratification of Individuals Who Underwent Epstein–Barr Virus-Based Screening for Nasopharyngeal Carcinoma: A Multicenter Cohort Study

Zhenhua Zhou, Tingxi Tang, Nan Li, Qiaocong Zheng, Ting Xiao, Yunming Tian, Jianda Sun, Longshan Zhang, Xiaoqing Wang, Yingqiao Wang, Feng Ye, Zekai Chen, Hanbin Zhang, Xiuting Zheng, Zhen Cai, Laiyu Liu* and Jian Guan**

Supporting Information

VLDL and LDL Subfractions Enhance the Risk Stratification of Individuals Who Underwent Epstein–Barr Virus-based Screening for Nasopharyngeal Carcinoma: A Multicenter Cohort Study

Zhenhua Zhou[1,2,†], Tingxi Tang[1,†], Nan Li[1,†], Qiaocong Zheng[1,3], Ting Xiao[1], Yunming Tian[1,4], Jianda Sun[1,5], Longshan Zhang[1], Xiaoqing Wang[1], Yingqiao Wang[1], Feng Ye[1], Zekai Chen[1], Hanbin Zhang[1], Xiuting Zheng[1], Zhen Cai[6 ‡], Laiyu Liu[2,‡], Jian Guan[1,7 ‡]

1. Department of Radiation Oncology, Nanfang Hospital, Southern Medical University, Guangzhou, Guangdong, China.

2. Chronic Airways Diseases Laboratory, Department of Respiratory and Critical Care Medicine, Nanfang Hospital, Southern Medical University, Guangzhou, Guangdong, China

3. Department of Radiation Oncology, Yangjiang People's Hospital, Yangjiang, Guangdong, China

4. Department of Radiation Oncology, Huizhou People's Hospital, Huizhou, Guangdong, China

5. Department of Radiation Oncology, Meizhou People's Hospital, Meizhou, Guangdong, China

6. Department of Laboratory Medicine, Nanfang Hospital, Southern Medical University, Guangzhou, Guangdong, China

7. Guangdong Province Key Laboratory of Molecular Tumor Pathology, Guangzhou, Guangdong, China

† These authors contributed equally to this work.

‡ Corresponding author: Jian Guan, Department of Radiation Oncology, Nanfang Hospital, Southern Medical University, Guangzhou, Guangdong, China; E-mail: guanj@smu.edu.cn; Zhen Cai, Department of Laboratory Medicine, Nanfang Hospital, Southern Medical University, Guangzhou, Guangdong, China; E-mail: caizh@smu.edu.cn; Laiyu Liu, Chronic Airways Diseases Laboratory, Department of Respiratory and Critical Care Medicine, Nanfang Hospital, Southern Medical University, Guangzhou, Guangdong, China; E-mail: laiyu@smu.edu.cn;

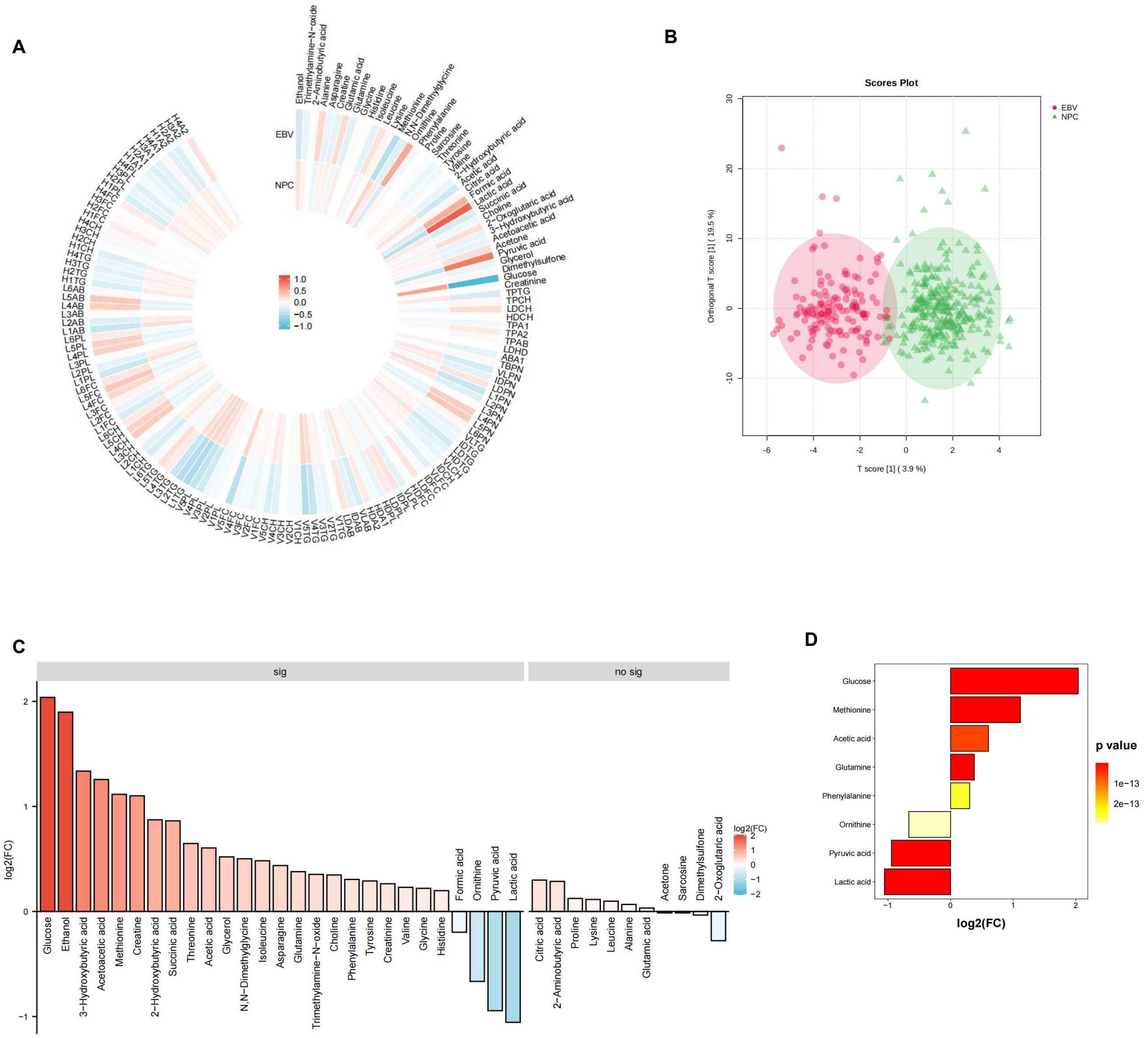

Fig.S1. Profile analysis of the plasma NMR metabolome. A: Circos plot of total metabolomic profiles of 150 plasma metabolites and lipoprotein subfractions; color represents the average expression abundance after standardization. B: Orthogonal PLS-DA score plot of total metabolomic profiles of the NPC and EBV cohorts; C: Log2-fold change in 38 plasma metabolites (NPC/EBV). D: Identification of signature metabolites with VIP > 1 and $p < 0.05$.

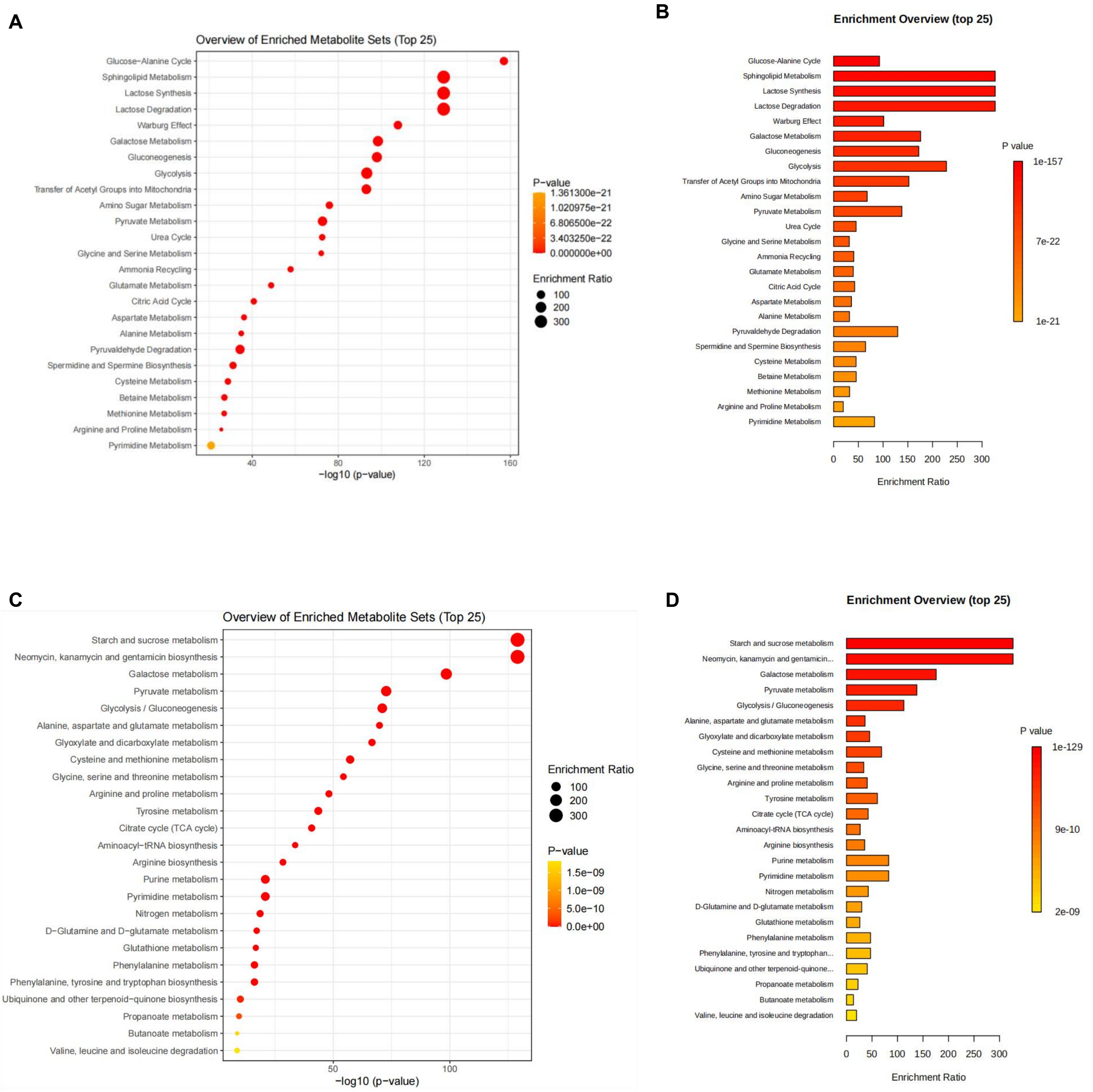

Fig.S2. Metabolite set enrichment analysis (MSEA) of the NPC and EBV cohorts. A: Dot plot of the enrichment analysis of 38 metabolite profiles in the SMPDB dataset (top 25); B: Enrichment ratio of the top 25 metabolic pathways in the SMPDB dataset (top 25); C: Dot plot of the enrichment analysis of 38 metabolite profiles in the KEGG dataset (top 25); D: Enrichment ratio of the top 25 metabolic pathways in the KEGG dataset (top 25).

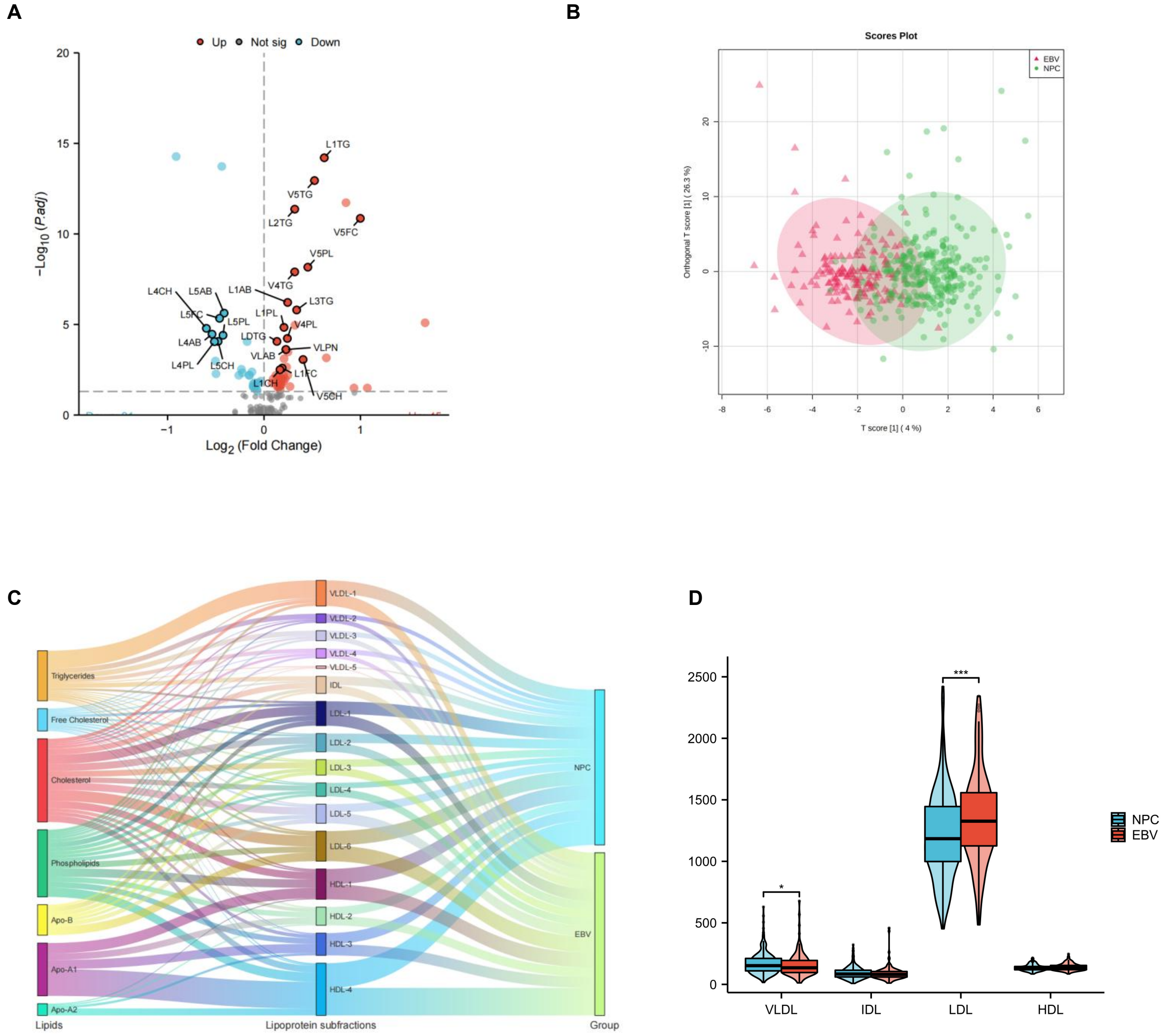

Fig.S3. Comprehensive analysis of plasma lipoprotein subfraction profiles. A: Volcano plot of 112 lipoprotein subfractions and 38 metabolites. B: Orthogonal PLS-DA score plot of 112 total lipoprotein subfraction profiles from the NPC and EBV cohorts. C: Sankey plot showing the differences in the composition of blood lipoprotein subfractions. D: Numbers of VLDL, IDL, LDL, and HDL particles in the NPC and EBV cohorts.

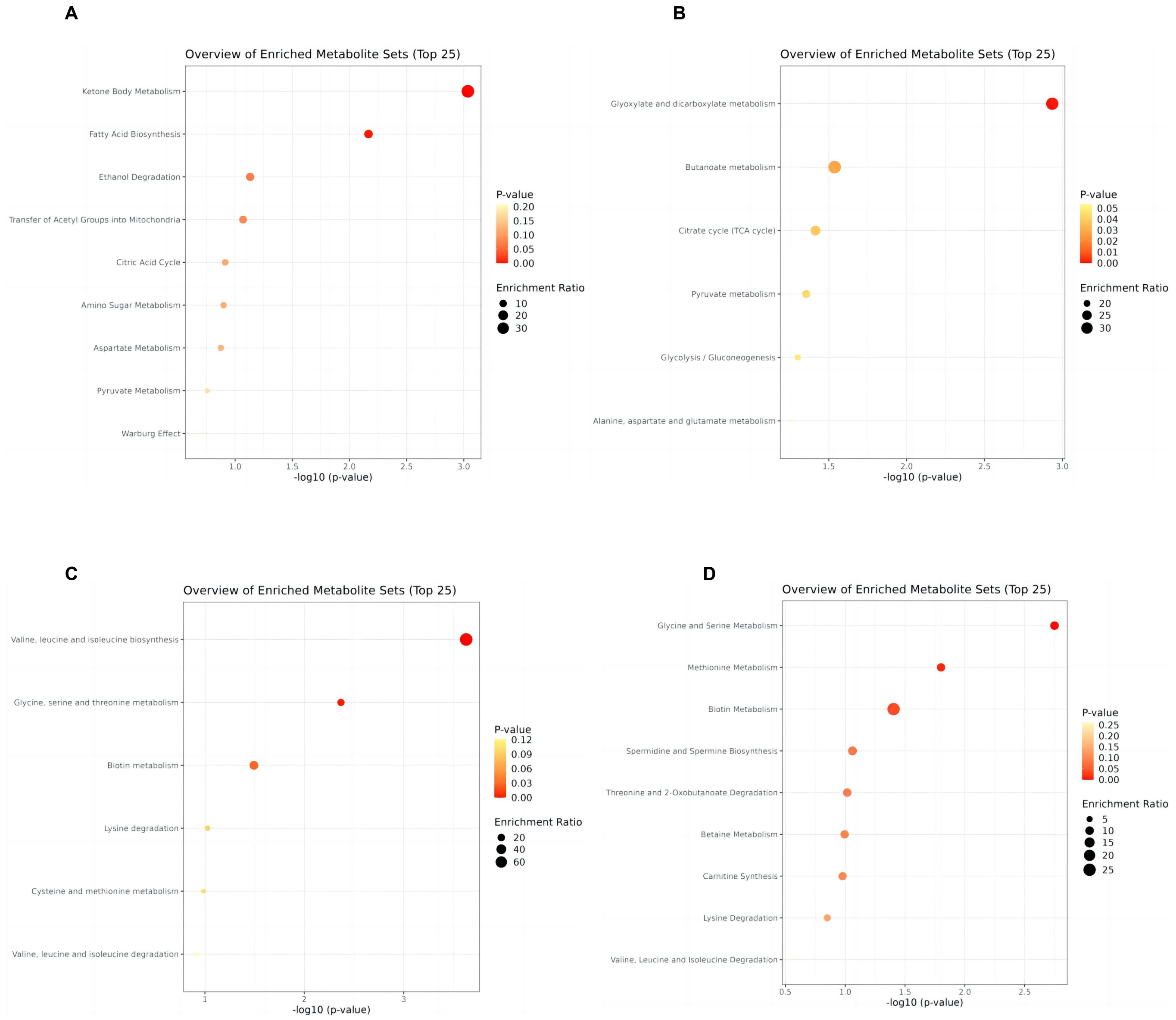

Fig.S4. Functional enrichment analysis of LDL-1 and VLDL-5 related metabolites. A: Dot plot of the enrichment analysis of LDL-1 related metabolites in the SMPDB dataset (top 25); B: Dot plot of the enrichment analysis of LDL-1 related metabolites in the KEGG dataset (top 25); C: Dot plot of the enrichment analysis of VLDL-5 related metabolites in the SMPDB dataset (top 25); D: Dot plot of the enrichment analysis of VLDL-5 related metabolites in the KEGG dataset (top 25);

Table S1. List of lipoprotein profiles.

	TG	CH	FC	PL	Apo-A1	Apo-A2	Apo-B	PN
**VLDL**	√	√	√	√	-	-	√	√
**IDL**	√	√	√	√	-	-	√	√
**LDL**	√	√	√	√	-	-	√	√
**HDL**	√	√	√	√	√	√	-	-
**VLDL-1**	√	√	√	√	-	-	-	-
**VLDL-2**	√	√	√	√	-	-	-	-
**VLDL-3**	√	√	√	√	-	-	-	-
**VLDL-4**	√	√	√	√	-	-	-	-
**VLDL-5**	√	√	√	√	-	-	-	-
**LDL-1**	√	√	√	√	-	-	√	√
**LDL-2**	√	√	√	√	-	-	√	√
**LDL-3**	√	√	√	√	-	-	√	√
**LDL-4**	√	√	√	√	-	-	√	√
**LDL-5**	√	√	√	√	-	-	√	√
**LDL-6**	√	√	√	√	-	-	√	√
**HDL-1**	√	√	√	√	√	√	-	-
**HDL-2**	√	√	√	√	√	√	-	-
**HDL-3**	√	√	√	√	√	√	-	-
**HDL-4**	√	√	√	√	√	√	-	-

TG, tryglicerides; CH, cholesterol; FC, free cholesterol; PL, phospholipids; Apo, apolipoprotein; PN, particle numbers; √, detected and quantified. -, not detected or not quantified.

The lipoproteins panel includes various fractions such as total HDL, LDL, IDL, and VLDL, along with 15 subfractions sorted numerically by increasing density and decreasing size: 4 HDL subfractions (HDL-1 to HDL-4), 6 LDL subfractions (LDL-1 to LDL-6), and 5 VLDL subfractions (VLDL-1 to VLDL-5). 8 additional parameters included TPCH, TPTG, TPA1, TPA2, TPAB, LDHD, ABA1,TBPN.

Table S2. List of density range of lipoprotein subfractions.

Lipoprotein	Density (kg L^{-1})
**VLDL**	0.950-1.006
**IDL**	1.006-1.019
**LDL**	1.019-1.063
LDL-1	1.019-1.031
LDL-2	1.031-1.034
LDL-3	1.034-1.037
LDL-4	1.037-1.040
LDL-5	1.040-1.044
LDL-6	1.044-1.063
**HDL**	1.063-1.210
HDL-1	1.063-1.100
HDL-2	1.100-1.112
HDL-3	1.112-1.125
HDL-4	1.125-1.210
